# LC–MS-Based Metabolomics Discriminates Premium from Standard Chilean cv. Cabernet Sauvignon Wines from Different Valleys

**DOI:** 10.3390/metabo11120829

**Published:** 2021-11-30

**Authors:** Vania Sáez, Doreen Schober, Álvaro González, Panagiotis Arapitsas

**Affiliations:** 1 Department of Food Quality and Nutrition, Research and Innovation Centre, Fondazione Edmund Mach, Via Edmund Mach 1, 38010 San Michele All’Adige, Italy; vaniastepsaezpulg@gmail.com; 2 Center for Research and Innovation, Viña Concha y Toro, Ruta K-650 Km 10, Pencahue 3550000, Chile; doreen.schober@conchaytoro.cl (D.S.); alvaro.gonzalez@conchaytoro.cl (Á.G.); 3 Department of Wine, Vine and Beverage Sciences, School of Food Science, University of West Attica, Ag. Spyridonos str, Egaleo, 12243 Athens, Greece

**Keywords:** *Vitis*, untargeted analysis, wine quality, pigments, tannins, sulfonation, indoles, peptides, resveratrol

## Abstract

Cabernet Sauvignon grapes in Chile, mainly grown between the 30° S and 36° S, account for more than 30% of Chilean wine production, and yield wines with different characteristics which influence their quality. The aim of this study was to apply a liquid chromatography – mass spectrometry (LC–MS)-based metabolomic protocol to investigate the quality differentiation in a sample set of monovarietal wines from eight valleys covering 679 km of the north-south extension. All samples were produced using a standardized red winemaking process and classified according to a company categorization in two major groups: premium and standard, and each group in two subcategories. The results pointed out that N-containing metabolites (mainly small peptides) are promising biomarkers for quality differentiation. Moreover, the premium wines were characterized by higher amounts of anthocyanins and other glycosylated and acetylated flavonoids, as well as phenolic acids; standard quality wines, on the other hand, presented stilbenoids and sulfonated catabolites of tryptophan and flavanols.

## 1. Introduction

The Cabernet Sauvignon grapevine cultivar produces iconic wines around the world and therefore covers about 4% (341,000 ha) of the global vine-cultivated area [1]. This wine grape variety was introduced in Chile around 1851 from Bordeaux before the phylloxera attacks on French vines [2]. Nowadays, Chile is one of the top Cabernet Sauvignon producers, with 13.6% of the global grape-growing area [3], which corresponds to 40% (40,204 ha) of the Chilean red grape production where 38,950 ha (96%) are between the Maipo and the Maule valleys [4]. The Chilean wine grape-growing area extends for approximately 1300 km of longitude in a narrow country with an average extension of 170 km between the Pacific Ocean to the west and the Andes mountain range to the east. Most of the vineyards are placed at a latitude of 30° S to 36° S, with the coastal range to the west strongly influencing the topography. The unique Chilean geography encompasses a wide range of different climatic conditions, from semi-arid according to the elevation in the north-central zone, to a dominant Mediterranean-type in the central zone [5,6], where variabilities in climate potential offer different characteristics for viticulture even in the same wine valley [6].

Wine, the result of the complete or partial alcoholic fermentation of fresh grapes, has an extraordinarily complex chemical profile, with innumerous metabolites that cover almost all the chemical classes of the primary and secondary plant metabolism, such as amino acids, lipids, peptides, carbohydrates, phenolics, organic and inorganic acids, indoles, amines, sulfur compounds, and volatile compounds [7]. These metabolites are present in a very wide range of concentrations (from g/L to ng/L) and, unfortunately, many of them are still unknown. The chemical diversity and complexity of the wine metabolome is the result of several required production steps that impact and are reflected in each bottle. More specifically, each wine metabolome is the result of many parameters, such as the vintage [8], the terroir [8,9], the grape phenotype [10], the cultivar [8,9], the addition of O_2_ and antioxidants such as SO_2_ [11,12,13,14], the fermentation process and yeast-related compounds [15], and the packaging and storage conditions [16,17]. All these steps influence the wine chemical signature and therefore its perceived quality. Indeed, by measuring the chemical compounds in a wine it is possible to obtain information and understand and explain its history, sensorial character, and quality.

Unraveling the association between the chemical composition (volatile compounds, non-volatile compounds, or both) of wine and its perceived quality is challenging and not straightforward. To pursue this objective, a few different targeted and untargeted research strategies and chemometric models have been implemented in wine research [8,10,18,19,20,21]. Sáenz-Navajas et al. (2015), by using a targeted approach in red wines, associated overall quality with higher levels of volatiles compounds such as norisoprenoids and lower levels of whisky lactones and volatile phenols, whereas the non-volatile compounds with astringent properties showed no correlation with quality [18]. For Italian Pinot noir wines, a positive correlation with wine quality was found for caftaric acid, quercetin 3-glucuronide, and glycerol, whereas gallic acid and delphinidin 3-glucoside showed a negative contribution to the overall wine score [21]. A reliable insight on wine chemistry in relation to quality can be achieved by targeted methods and multivariate statistics, but due to the high complexity of the wine metabolome, this is a limited approach, because the relevant strategies miss a high number of metabolites. On the other hand, an unbiased strategy with a holistic approach is more promising for discrimination purposes in terms of quality [8,20].

Using a comprehensive analysis with an untargeted approach, the latest research on commercial wines demonstrated that the non-volatile compounds could describe the wine quality assessment by wine experts better than volatile compounds [20]. In commercial monovarietal Chilean wines, it has been possible to discriminate according to quality score using non-volatile compounds [8]. For Pinot noir wines, quality was positively associated with dipeptides and unsaturated fatty acids, whereas N-(3-methylbutyl)-acetamide and xanthine were negatively associated with quality using an untargeted approach [20]. The chemical fingerprint from Pinot noir wines from three different grape clones was associated to their sensorial properties and their quality. The results show associations between non-volatiles compounds, such as anthocyanins and derivatives, with aroma perception and between anthocyanins, flavonol, and flavanol compounds with sweetness, bitterness, acidity, and astringency and their quality [10].

Within this framework, a research project was designed to investigate the metabolome of a sample set composed of 150 well-defined Chilean Cabernet Sauvignon single vineyard experimental wine samples (50 × 3 biological replicates), originated from grapes harvested from eight Chilean valleys and produced under the same semi-industrial standardized red winemaking process. A robust LC–MS-based untargeted/metabolomics workflow was chosen for the acquisition of their chemical fingerprint. The initial aim was to find tentative metabolite markers of their quality classification based on a commercial categorization. These markers should allow us to understand the Chilean Cabernet Sauvignon wine (sub)qualities, find the biosynthetic pathways and chemical reactions that are responsible for their differentiation/classification, make new hypotheses to be validated in future projects, and create new knowledge for Chilean winemaking.

## 2. Results

### 2.1. Standard versus Premium Wines

**Figure 1:**
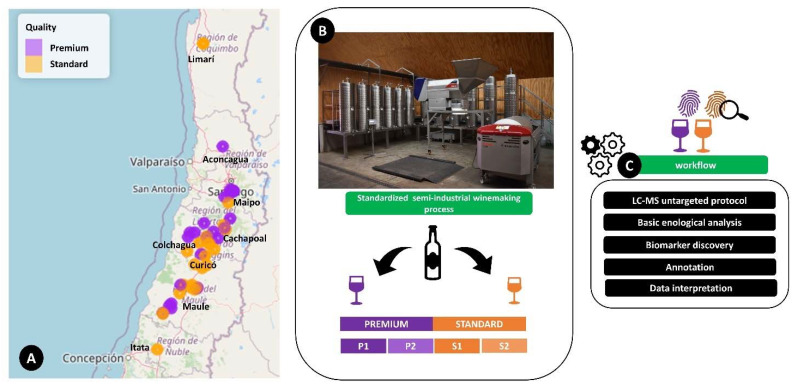
Schematic representation of experimental design of the study, (**A**) grape distribution of sampled vineyards in different Chilean valleys according to quality group, (**B**) winemaking and classification for experimental wine samples (P1: super-premium, P2: premium, S1: standard plus, and S2: standard), (**C**) schematic data acquisition and analysis workflow.

Grapes were harvested from eight different valleys (Limarí, Aconcagua, Maipo, Cachapoal, Colchagua, Curicó, Maule, Itata) as represented in Figure 1. This sample distribution in our experimental design allowed us to cover approximately 679 km of longitudinal distance (Supplementary Material, Figure S1). Additional information on grapes, grape musts, and the conduction trellis systems of the specific vineyards is available in Table S1.

**Figure 2:**
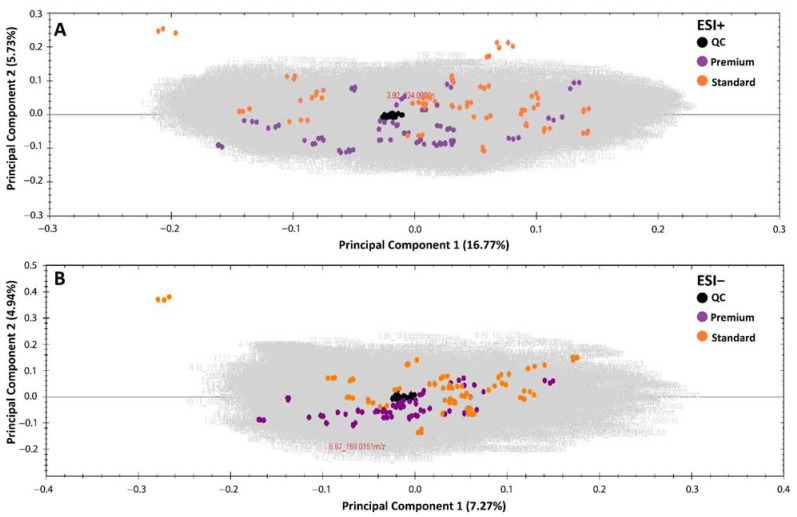
PCA plots of all features registered in Cabernet Sauvignon wine samples in ESI+ (**A**) and ESI− (**B**).

The resulting grape must have an average of 23.3 ± 2.1 Brix, whereas the resulting experimental wines had a mean pH of 3.4 ± 0.1, an alcoholic content of 13.2 ± 0.8%, a concentration of total sulfur dioxide (TSO_2_) of 46.3 ± 5.4 mg/L, and a concentration of free sulfur dioxide (FSO_2_) of 39.9 ± 5.3mg/L; the only difference between the wine groups (*p*-value ≤ 0.05) was in the FSO_2_ concentration, where the standard wines had a higher concentration (41.7 ± 5.3 mg/L) than that of the premium wines (38.3 ± 4.8 mg/L). All the basic oenological analysis data are available in Table S2. Before performing further analyses, the quality of the data must be assessed. The reliability and quality of the data acquired in a single batch were assessed by a principal component analysis (PCA) for each ionization mode. As shown in Figure 2, the QC injections, both for ESI+ and ESI−, clustered together, as did the three biological replicates of each wine sample. This is fundamental in order to guarantee the robustness of the data set and thus for the results and the tentative biomarker discovery. The number of features registered in the unsupervised analysis was 9258 in ESI+ and 7633 in ESI− (Figure 2)

**Figure 3:**
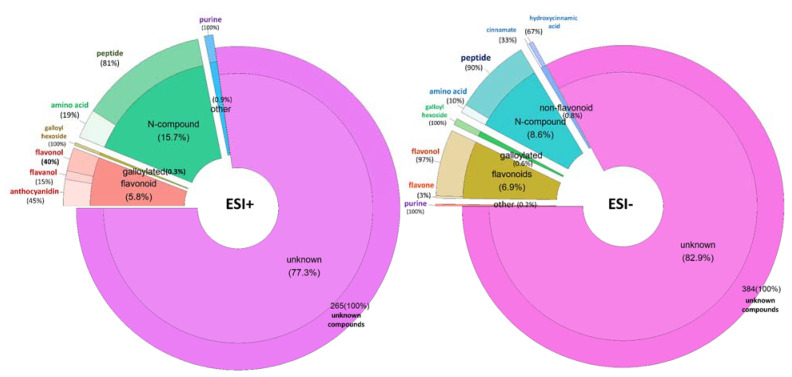
Percentage of biomarkers according to their chemical classification for discriminate premium and standard Cabernet Sauvignon wines by ESI− and ESI+.

According to the quality company categorization, 25 experimental wine samples were classified as premium quality and 25 as standard quality (Table S2, Figure 1). To investigate the differences between the metabolomic fingerprints of the wine groups, an ANOVA analysis was performed. The putative biomarkers selected (features with *p*-value ≤0.01, maximum fold rate ≥ 2) for each group were 343 compounds and 463 in ESI+ and ESI−, respectively (Tables S3 and S4). As shown is Figure 3, the highest percentage of annotated tentative biomarkers for both ionization modes, which allowed us to discriminate the standard from premium wine groups, comprised N-containing compounds, mostly short-chain peptides. The other annotated tentative biomarkers belonged to other chemical classes, mainly polyphenols, flavonols, anthocyanins, and hydroxycinnamic acids. Initially, we were surprised by the high number of N-containing compounds that resulted as tentative markers for both the ESI+ and ESI− mode. Nitrogen is an essential nutrient, able to affect yeast cell growth and fermentation kinetics during wine alcoholic fermentation, and *Saccharomyces cerevisiae*, even though it prefers to use simple inorganic nitrogen as free amino acids and ammonium, in some conditions is able to use amino acids, peptides, or oligopeptides and proteins as secondary nitrogen sources [22]. According to the basic oenological analysis, the content of yeast assimilable nitrogen (YAN), primary amino nitrogen (PAN), and ammonium (NH_4_^+^) measured in the fresh grape musts of the Cabernet Sauvignon samples did not show significant differences between the premium and standard wine groups (Table S7). Since all the wines were produced under the same winemaking semi-industrial standardized procedure, with the yeast strain being the same for all fermentations, it is highly probable that the differences in N-containing compounds were caused by the starting material composition (grapes from different vineyards and valleys).

### 2.2. Super Premium versus Premium versus Standard plus versus Standard Quality Wine Groups

For a better understanding of the metabolomic differences in the Cabernet Sauvignon experimental wines, we decided to continue the marker investigation based on four commercial quality subclasses. The sub-classification of wine samples was made according to the company categorization of the expected quality for a premium or standard Chilean Cabernet Sauvignon wine (Table S2). The 25 premium samples were divided into super-premium P1 (7 samples) and premium P2 (18 samples), whereas the 25 standard samples were divided into standard plus S1 (15 samples) and standard S2 (10 samples).

700 features with a *p*-value ≤ 0.01 and max folder ≥ 2 in ESI− and 1563 in ESI+ were putative biomarkers for the quality subgroups. From the selected features, 59 biomarkers (*p*-value ≤ 0.01 and max folder ≥ 2) were annotated with different levels of confidence (most of them at level 3). More than 90% of the selected features annotated for group discrimination were unknowns, which demonstrates the wine metabolomic complexity. The annotation was carried out semi-manually by using an internal library of chemical references, analyzed under the same instrumental conditions (1st level annotation), and external databases or literature [9,11,17,23,24]. Additionally, we included compounds with enological importance (markers) with a *p*-value ≤ 0.05 in the annotated compounds list. The biomarker discovery and annotation steps produced a list of 115 compounds tentatively annotated as biomarkers and markers. The full list of these annotated compounds includes amino acids, flavonoids, phenolic acids, cinnamates, sulfonates, lipids, vitamins, purines, stilbenes, etc.

**Figure 4:**
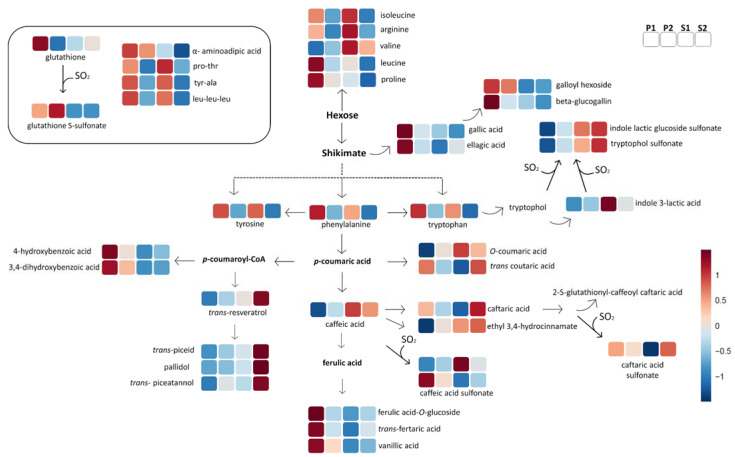
Schematic representation of biosynthesis and synthesis for the wine quality groups (P1, P2, V1, and V2) of N-containing metabolites and non-flavonoids (benzoic acids, hydroxycinnamates, hydroxycinnamic acids, and stilbenoids). The color code refers to the heatmap available in the Supplementary Information (Figure S4).

**Figure 5:**
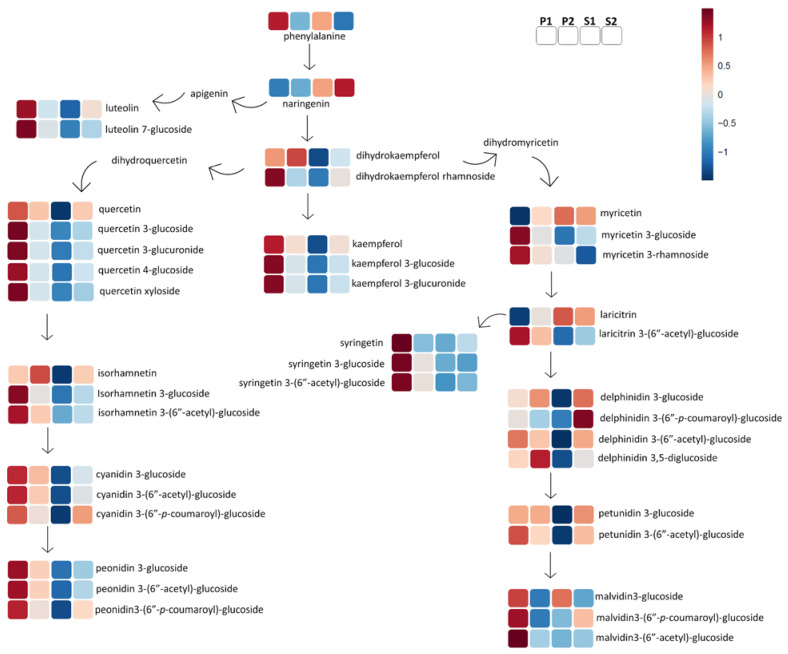
Diagram of flavonoid biosynthesis and synthesis of flavanone, flavanols, and anthocyanins for wine quality groups (P1, P2, S1, and S2). The color code refers to the heatmap available in the Supplementary Information (Figure S4).

**Figure 6:**
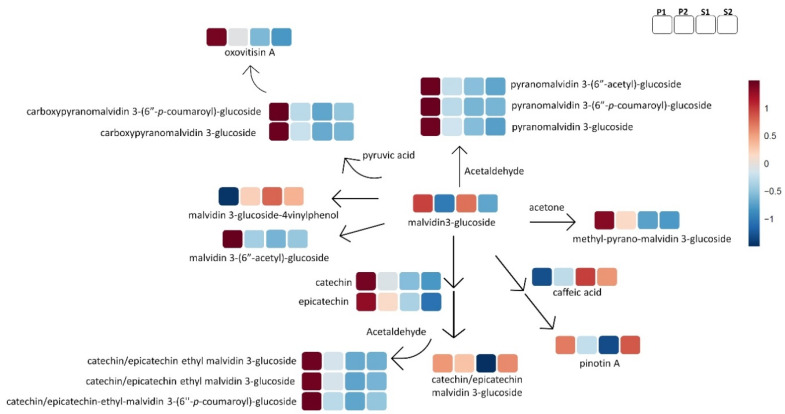
General schematic representation of wine pigments pattern by wine quality group (P1, P2, S1, and S2). The color code refers to the heatmap available in the Supplementary Information (Figure S4).

**Figure 7:**
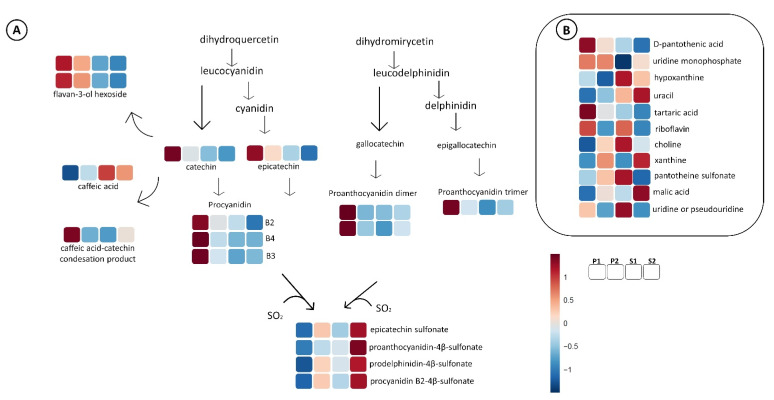
(**A**) General representation for P1, P2, S1, and S2 flavanol patterns for the sulfonation reaction in the flavanols. (**B**) Diagram for the marker compounds, such as purines or vitamins. All the metabolites in Figure 7 are markers (*p*-value ≤ 0.05) for wine quality subgroups. The color code refers to the heatmap available in Figure S4 (Supplementary Information).

The next step was to turn back to the raw files and make a semi-manual peak integration of all the above tentative markers, and we included other metabolites that belong to the same biosynthetic chemical group pathway as the biomarkers and were previously annotated with the same analytical protocol. The output of this process delivered a table with 163 compounds with enological importance (Table S6), with the integrated areas of annotated compounds (Table S7), which was uploaded to the online platform at http://www.metaboanalyst.ca (accessed on 9 November 2021) for further statistical analysis, and the information produced by the platform heatmaps was used to create the pathway illustrations of Figure 4, Figure 5, Figure 6 and Figure 7.

#### 2.2.1. Amino Acids and Peptides

Generally, the subclasses P2 and S2 were less rich in free amino acids in comparison to the P1 and S1 wines (Figure 4 and Table S11). More specifically, the P1 was characterized by proline and leucine, and the levels of tryptophan, tyrosine, and phenylalanine were higher in P1 and S1. The metabolomic pathway of tryptophan in grapes includes the biosynthesis of the indole 3-lactic acids and their glucoside, and the biosynthesis of tryptophol in yeasts [13,25]. All these tryptophan products can react with SO_2_ in wine, leading to the formation of their sulfonated analogs [12,13]. The S1 wine group shows a higher concentration of indole 3-lactic acid than the other wine quality groups. The concentration of indole 3-lactic acid glucoside may vary due to genetics [9,25] or wine style [13]. According to our experiment, indole 3-lactic acid glucoside levels were influenced by the valley of origin (Figure S2) but were not important in discriminating wine quality (Tables S6 and S11). Moreover, we noticed that the sulfonated derivatives of tryptophol and indole 3-lactic acid showed a significantly higher concentration in the two standard quality groups, S1 and S2. Surprisingly, there were no significant differences between the four quality groups in terms of measured levels of free or total SO_2_ (Table S10).

Glutathione, a tripeptide, antioxidant, and *O*-quinone scavenger with a characteristic thiol moiety, is naturally present in wines [7]. One of the characteristic markers for premium wines (P1 and P2) is the sulfonate analogue of glutathione: S-sulfonate glutathione, which was firstly described in white wines and lately in red wines [12,17]. This short peptide is a product of the sulfitolysis of oxidized glutathione, while the chemical reaction for their formation is prompted by higher amounts of oxygen [17].

Other N-containing metabolites, such as α-aminoadipic acid, Leu-Leu-Leu, hydroxyproline, Gly-His, Tyr-Ala, and Pro-Thr (Figure 4), were annotated with the tentative biomarkers. To the best of our knowledge, this is the first time that the Leu-Leu-Leu, Gly-His, Tyr-Ala, and Pro-Thr peptides have been identified in red wines, and their annotation was confirmed with their corresponding commercial reference (1st level annotation). The metabolite α-aminoadipic acid was a characteristic marker for the premium groups (P1 and P2).

#### 2.2.2. Non-Flavonoid Polyphenols

Figure 4 shows a schematic description of the shikimate pathway part that delivers hydroxycinnamic acids, benzoic acids, and stilbenoids, and at the same time the behavior of these metabolites in the various wine subclasses. The lower quality subclasses of wines in our experiment design (S2) were characterized by high levels of stilbenoids (*trans-*resveratrol, *trans-*piceid, *trans*-piceatannol, and pallidol); at the other end of the spectrum, the higher quality wines (P1) were characterized by high concentrations of phenolic acids (mono-, di-, and tri-hydroxybenzoic acids), ellagic acid, vanillic acid, and ferulic acid derivatives. Two of the stilbenoids annotated in our study as quality markers—*trans*-piceatannol and pallidol—did not show differences between valleys (Table S12).

In addition, the standard quality wines (S1 and S2) showed a tendency to be characterized by the not methylated hydroxycinnamate derivatives, such as caffeic acid and caftaric acid. Another good biomarker for P1 groups in our experiment was tentatively annotated as galloyl hexoside (Figure 4).

#### 2.2.3. Flavonoids

Figure 5, Figure 6 and Figure 7 provide a general overview of the comparison of the four wine quality groups based on the concentration and synthesis of flavonols, monomeric and oligomeric flavan-3-ols, and anthocyanins and related pigments, a set of phenolic compounds with paramount importance in the description of wine quality, sensorial character, and bioactivity.

The premium groups were characterized by the highest concentration in almost all flavonols (quercetin, kaempferol, myricetin, syringetin, laricitrin, luteolin, and isorhamnetin). In particular, the P1 wine group was characterized by the highest levels of flavonol derivates such as acetylated flavonols or glycoside (Figure 5 and Table S13). Three acetylated flavonols are promising markers for wine with the highest quality and were tentatively annotated as isorhamnetin 3-*O*-(6′-acetyl)-glucoside, laricitrin 3-*O*-(6′-acetyl)-glucoside, and syringetin 3-*O*-(6′-acetyl)-glucoside (Figure 5), according to their MS fragmentation patterns (Table S5) as identified by Favre et al. [26]. To our knowledge, this is the first time this kind of compound has been detected in Chilean wines.

Regarding the anthocyanins, it is possible to identify a pattern for premium wines in the B-ring substituted cyanidin and peonidin series (3-*O*-glucoside, 3-(6′-acetyl)-glucoside and 3-*O*-(6′-*p*-coumaroyl)-glucoside); this anthocyanin trend does not apply to the delphinidin, petunidin, and malvidin series (Table S13), with the exception of the biomarker for the premium wines: delphinidin 3,5-*O*-diglucoside (Table S3). The group with the poorest anthocyanins profile was also the poorest in flavonols, namely, S1.

During winemaking and aging, anthocyanins react with other metabolites and deliver various new pigments, such as the direct or ethyl-bridge-linked flavanol-anthocyanins (F-A), the pyranoanthocyanidins, the carboxypyranoanthocyanidins, and the hydroxyphenylpyranoanthocyanidins [7]. Such reactions are very important for color stability or loss during aging, and are fundamental to explaining wine quality. Figure 6 includes several of these red pigments, highlighting that the P1 group was by far the richest one in this metabolite group. This trend should be a key parameter to explain why historically the P1 wine group is categorized with the highest quality in comparison to the other three quality groups. Of course, we need to underline that the two hydroxyphenylpyranoanthocyanidins with a characteristic yellow-orange nuance included in Figure 6 do not follow this trend: malvidin 3-glucoside-4-vinylphenol has not shown any difference between the various quality groups, while pinotin A is present in higher concentrations in the S2 group and lower in the S1. Finally, the directly linked F-A pigments had similar concentrations in all groups, apart from the S1 group, where it was much lower.

Figure 7A is dedicated to the monomeric and oligomeric flavanols (dimer and trimer). The premium P1 group had the highest concentration in most of this polyphenolic class, and the two premium groups (P1 and P2) had higher concentrations in epicatechin than standard quality wines.

The monomeric flavan-3-ol hexoside (Figure 7A and Table S6) was also tentatively annotated in the Chilean Cabernet Sauvignon wines. The fragmentation pattern in ESI−mode for the compound annotated as 451.1246 *m/z* flavan-3-ol hexoside [M-H]^−^, had a loss of glucoside moiety (162 *m/z*) with the formation of 289 *m/z* and a subsequent loss of 44 Da (CO_2_) by decarboxylation, generating 245 *m/z*, which clearly indicated catechin/epicatechin in the structure core (Table S5). This kind of monomer with hexoside substitution has been reported in different wine varieties [27]. The levels of flavan-3-ol hexoside compounds were higher in P1 wines and thus are a characteristic marker for premium wines in general. To the best of our knowledge, this is the first time the flavan-3-ol hexoside monomers have been reported in Chilean wines.

The group with the lowest quality (S2), had the highest content of sulfonated tannins (Figure 7A). This class of tentative marker metabolites includes both monomeric (epicatechin 4-sulfonate) and dimeric (proanthocyanidin sulfonate, procyanidin B2 4-sulfonated, and prodelphinidin sulfonate) sulfonated flavonols. On the opposite end of the spectrum, the wine group with the highest quality (P1) was the poorest in sulfonated flavonols. It was recently demonstrated that this sulfonation reaction is promoted by high temperatures and low pH, and that their synthesis in wine involves the depolymerization of tannins [28]. The wine samples in the present study had the same industrial winemaking protocol, aging time, and storage conditions, so the reaction with bisulfite in the S2 wine group must be promoted or triggered by another unknown factor, which could be related to the raw material and its geographical origin.

Some sulfonated analogs of procyanidins and indoles were negatively correlated with pyranoanthocyanidins, carboxypyranoanthocyanidins, and ethyl bridge anthocyanin-flavanols (Figure S3). The premium wines which had the higher levels of ethyl bridge anthocyanin-flavanols, pyranoanthocyanidins, and methylpyranomalvidin 3-*O*-glucoside also had the lowest levels of sulfonated procyanidins and tryptophol sulfonate, and the opposite trend was shown for standard wines in our study (Figure 6 and Figure 7A). This strongly suggests a different oxidative status for the semi-industrial experimental wines, underlining a possible reaction(s) indirectly promoted by the availability of O_2_ and/or SO_2_ in the winemaking phase or the wine environment and, consequently, the release/accumulation of acetaldehyde or hydroxyethyl sulfonate which in turn may modify the initial wine profile. Undoubtedly, it is necessary to obtain a deeper understanding of the key conditions or factors impacting the grapes as a raw material and promoting this reaction(s), in order to better understand their impact on wine quality.

#### 2.2.4. Other Compounds

Figure 7B includes some annotated tentative markers that do not belong to any of the above-described classes. Hypoxanthine was positively correlated with standard quality wines (S1 and S2) and xanthine with the S2 and P2 groups, the lowest quality for each category. Other tentative markers, which characterized the premium wines, were uridine monophosphate and D-pantothenic acid.

## 3. Discussion

Our results indicated that the N-containing compounds annotated as peptides were highly important tentative biomarkers to differentiate premium from standard Chilean Cabernet Sauvignon wine quality. This is not the first time that N-containing compounds and especially small peptides have been found to be responsible for the quality differences in wines [20]. In addition, peptides in fermented foods are taste-active (some are also bioactive) compounds [29,30,31], promoting healthy effects and being highly susceptible to modifications during food processing due to the presence of exposed active groups in their amino acidic residues [32]. In wine they are tentatively associated to certain sensorial attributes, such as sweetness [33], bitterness, and sourness, and γ-glutamyl peptides contribute to the umami taste in fermented foods [31,33,34]. More recently, dipeptides have been reported to contribute to the perceived quality of commercial Pinot noir [20] and N- and S-containing compounds similarly contribute to Chardonnay wines [35]. The results of our research underline the need to investigate further the role of peptides in wine quality and in their perception.

Indoles are markers for standard wines groups (Figure 4) and might have an influence on wine quality, as they are associated with *off-flavors* or “atypical aging” by indole degradation products [36]. In addition, indole 3-lactic acid, a biomarker for the S1 wine group, was recently identified as an important metabolite for quality prediction in Pinot noir wine [20].

One marker for premium wines is the α-aminoadipic acid; this metabolite is a ketoglutarate-derived intermediate of lysine biosynthesis [37] or a product of lysine oxidization when exposed to reactive oxidative species (ROS), which can affect susceptible amino acids in food production, processing, or storage [38]. In grape must in fermentation, α-aminoadipic acid is suggested as a possible second source of nitrogen for yeast strains with a different assimilation mode when other preferred nitrogen sources are depleted; this is related to the uptake of glutamate-rich peptides by oligopeptide transport under physiological control modulation [37].

Recently, one of the metabolites associated with the spice attribute in Chilean Pinot noir wine was tentatively annotated as galloyl hexoside [10], which also turned out to be a tentative biomarker for the P1 group in our experiment (Figure 4). A marker for the S2 group was annotated as xanthine. This metabolite was identified as a marker in two untargeted LC–MS based projects, the first on Sangiovese wines oxidation [11] and the second related to low-quality Pinot noir wines [20].

The sulfonation reaction mechanism(s) is the key for the subdivision of wine groups according to quality. The results show that sulfonated flavanols as well as sulfonated tryptophol catabolites are characteristic in standard wines (S1 and/or S2), while in the case of premium wines, glutathione is preferred to bisulfite anion as a substrate (Figure 4 and Figure 7). The sulfonated tryptophan catabolites were described for the first time in white wines and were positively correlated with high oxygen levels in the headspace [12] but were later detected in red wines as well [9,12,17]. Their impact on the wine sensorial character and its perceived quality is still unknown but could be correlated first with the formation of 2-aminoacetophenone (2AA), a compound indicated as being mainly responsible for the *off-flavor* [36], and second with the bitter taste in wine [39,40]. Regarding sulfonated proanthocyanidins which are markers for the S2 wine group, they are associated with “in house” sub-optimal storage [16], anoxia [17], wine style and aging time [13,14], and origin [9]. The possible correlation between the sulfonated flavanols and a wine’s astringent character was recently hypothesized in a study that included both chemical and sensorial analysis in a large sample set of red wines [9,41]. It was strongly suggested that tannin sulfonation may influence the declination of astringency perception in red wines, probably by reducing the binding of tannin-protein in salivary proteins [14]. S-sulfonated glutathione is a biomarker for premium wines; glutathione is known as antioxidant and “*kokumi*” compound, a flavor enhancer for umami, a sweet and salty taste in foods [7,34]. There is lack of information on whether S-sulfonated glutathione preserves the antioxidant or flavor-enhancing properties of glutathione. In our experimental design the oxygen level was not a variable in aging and the wines were opened in a N_2_-inert environment for analysis. This suggests that there are more mechanisms or unknown factors related to grapes and their geographical origin that promote the sulfonation and affect the wine quality. A possible oxidation mechanism triggered by unknown or not-well-characterized factors in wines with different qualities is suggested, due the negative correlation between sulfonated procyanidins and tryptophol catabolites and compounds reacting with acetaldehyde, a main oxidative byproduct.

Benzoic acids and hydroxycinnamates were more characteristic of premium wines (Figure 4), while the lowest quality standard wines in our study were well characterized by stilbenoids. Phenolic acids are associated with the enhancement of the astringency when they are mixed with other sensorial active astringency compounds, such as flavanols [42]. Stilbenoids gained attention when the “French paradox” was published, correlating moderate wine consumption with a low incidence of cardiovascular disease, despite a diet rich in high saturated fatty acids [43]. The stilbenoids concentration in grapes can be affected by genetic, biotic, and abiotic factors (cultivar, clone, micro- and macro-climate, viticulture practices, etc.), or can be produced by the plant in response to attacks by pathogens, such as *Botrytis cinerea*, *Plasmopara viticola*, and *Aspergillus* spp. [44]. Therefore, a possible explanation of the high content in stilbenoids for the S2 group could be that they were produced by the vine as a response to a biotic or abiotic stress, or a combination of both. The information regarding a direct effect of stilbenoids on the wine sensorial character is very limited and is mostly focused on the *trans*-resveratrol monomer. It is reported that in Cabernet Sauvignon wines enriched with *trans*-resveratrol, there is no alteration of the flavor or aromatic attributes [45], whereas in the Blaufränkisch wine variety fortified with *trans*-resveratrol, the intensity of the odor and color improved [46]. Very recently, piceid was associated with coffee/chocolate attributes in Pinot Noir wines [10]. Certainly, the stilbenoids are relevant biomarkers in our research to describe the wine groups, but further research is needed to understand the role of stilbenoids as quality markers.

Regarding flavonoids, the glycosylated and acetylated glycoside flavonol and some anthocyanins such as cyanidin and peonidin series are biomarkers or markers for premium wine (Figure 5 and Figure 6). This result can highlight a different acetyl and glycosyl transferase activity in the raw material between quality wine groups which influences the wine. The premium group P1 had the highest level of anthocyanins and anthocyanin pigments. Recent research suggests the anthocyanins and anthocyanin pigments profile is relevant beyond the color stability in wines [10,42,47]. Anthocyanin showed a role in the mouthfeel sensorial “bitter” and “dryness” dimension, but this effect can be masked by other wine metabolites [47]. Additionally, the anthocyanins in particular glycosides and pyranoanthocyanins can have an indirect astringent sensorial effect via protein precipitation, due to their affinity for salivary proteins [42,48], and glycosides are associated with contributing to aroma attributes in Pinot noir wines despite being described as odorless compounds [10]; these properties can have an impact on wine quality, but the sensorial effect of anthocyanin is chiefly indirect and probably wine matrix-dependent [47]. The super-premium group had the richest anthocyanin profile by far, but further research is necessary to obtain a deeper understanding of the anthocyanins’ role in the perceived wine quality and their interactions according to the wine metabolomic profile beyond the attractiveness of color in premium wines.

It is possible the conditions of the vineyards in different valleys producing premium wines favor the biosynthesis of all the above-mentioned metabolites, which are crucial for the color and the taste of the produced wines. On the other hand, the Chilean vineyards that produced standard quality Cabernet Sauvignon were characterized by wines rich in stilbenoids, sulfonated indoles, and sulfonated flavanols. A possible explanation could be that the biotic and abiotic conditions of these vineyards pushed the vine to produce more stilbenoids over the production of other polyphenols (tannins, flavonol, flavanol, anthocyanins, etc.), and later the produced wines are more sensitive to the reactions with SO_2_ in winemaking or aging, which can directly impact the wine quality.

One research limitation is the fact that all samples came from the same vintage (2018). Due to the metabolomic complexity, further research on wines from other vintages is needed to prove our hypothesis and obtain better insights of the Chilean Cabernet Sauvignon wine fingerprint profile according to wine quality.

## 4. Materials and Methods

### 4.1. Winemaking Procedure

The winemaking process to produce experimental monovarietal wines was performed using a standardized red winemaking protocol in the experimental winery of the Center for Research and Innovation (CRI)—Viña Concha y Toro, Pencahue, Chile. The Cabernet Sauvignon grapes were harvested from different Chilean valleys in 2018. The basic oenological characteristics and geographic information of the vineyards are available in the Supplementary Information section (Table S1).

For the winemaking process, 600 kg of harvested grapes were received in the experimental winery. The winemaking process was performed in 5 days. The grapes were destemmed, crushed, and transferred to a 1-ton fermentation bin, stabilized with 5 g of SO_2_ per hL, and adjusted to 23.5 °Brix and a pH of 3.45. For alcoholic fermentation, yeast was added (20 g/hL, Maurivin PDM, AB Biotek, Peterborough, UK), together with diammonium phosphate. The fermentation was conducted at 24–26 °C. In this step, a second dose of diammonium phosphate (60 mg/L) was added. On the third day of fermentation, 8 mg/L of O_2_ was added_,_ and the measure density was 1060 g/L (densimeter Alla France, Chemillé, France). The end of alcoholic fermentation on the fifth day was confirmed by measuring the residual sugar with an enzymatic D-glucose/D-fructose reagent kit in a Y15 automatic analyzer (Biosystem, Barcelona, Spain). The alcoholic fermentation process concluded with a level of residual sugar of <2 g/L. The wine was transferred to stainless steel tanks of 100 L, and pH was adjusted to 3.5 before malolactic fermentation was conducted at a temperature of 20–22 °C (MF, 4 g/HL, Viniflora CH 16, Christian Hansen, Hoersholm, Denmark). Wines were kept in the tanks under 1.5 bar N_2_ for approximately one month more, and were thereafter filtered, had their SO_2_ levels adjusted to 35 mg/L, were bottled in dark green glass, and were stored at 12 °C until further analysis.

### 4.2. Chilean cv. Cabernet Sauvignon Wines Samples

Fifty monovarietal experimental wines (produced in triplicate for a total of 150 samples bottles), vinified as described in the above section, were used in the study. The wines were classified according to the company categorization, based on the historical quality of the specific vineyard and the sensory evaluation performed by winemaking experts [49] in two main categories: 25 wines were classified as premium (P) and 25 wines as standard (S). The basic oenological information on the wines is provided in the Supplementary Information section, in Table S2. The premium category was divided in to “super-premium” quality (P1), with 7 wine samples and “premium” quality (P2), with 18 samples, whereas the standard category was divided into “standard plus” quality (S1), with 15 samples, and “standard” quality (S2), with 10 wine samples. The number of samples according to origin were as follows: 2 samples from Limarí, 8 samples from Maipo, 10 samples from Colchagua, 6 samples from Cachapoal, 15 samples from Maule, 7 samples from Curicó, 1 sample from Itata, and 1 sample from Aconcagua (Figure 1).

### 4.3. LC–MS-Based Metabolomics Analysis

A previously described and robust protocol was used for the acquisition of the wine metabolomic space [50]. The wine bottles were opened in April of 2019 in a N_2_-environment to avoid triggering or promoting undesired reactions, and 1 mL of each wine sample was pooled for the quality control (QC) sample. The 150 wine bottles were aliquoted in 10 mL dark vials by completely filling the vial. The sequence, as well the preparation order of the samples, was according to a randomized list. A total of 2 mL of sonicated Milli-Q water was added to 1 mL of the wine samples (including the QC), which was then filtered with 0.22 µm PVDF filters into a dark LC–MS vial.

The UPLC-QToF MS analysis was conducted according to the published protocol [9]. A Waters Acquity UPLC (Waters, Manchester, UK) was used in tandem with a Synapt HDMS QTOF MS (Waters, Manchester, UK) controlled by MassLynx^TM^ 4.1 software (Milford, MA, USA). An Acquity UPLC 1.8 µm, 2.1 × 150 mm, HSS T3 column (Waters) was used for the chromatographic separation at 40 °C with a flow of 0.28 mL/min for 5 µL of injected sample. The mobile phases were 0.1% of formic acid in water (mobile phase A), and in methanol (mobile phase B), respectively. The gradient was as follows: for the first minute, 0% of B; then 0–10% of B (1–3 min); 10–40% of B (3–18 min); 40–100% of B (18–21 min); 100% of B kept for 4.5 min (until 25.5 min of gradient); then 100–0% of B (25.6–28 min). The MS data were acquired in positive (ESI+) and negative (ESI−) mode, in W mode in a mass range of 50–2000 amu in centroid mode, with a scan duration of 0.4 s. The transfer collision energy and trap collision were settled with the values of 6 V and 4 V, respectively. For the source parameters the capillary for ESI+ and ESI− was slightly different, 3 kV for ESI+ and 2.5 kV for ESI−; meanwhile, for each mode scan the values were the same for the sampling cone (25 V), extraction cone (3 V), source temperature (150 °C), desolvation temperature (500 °C), and flow for the nebulizer (50 L/h) and desolvation gas (1000 L/h). A leucine enkephalin solution was used (0.5 mg/L in 50:50 of methanol water with 0.1% of formic acid) at 0.15 mL/min for lock mass calibration, whereas a sodium formiate solution (0.1 M of NaOH/10% formic acid/in acetonitrile at a ratio 1:1:8) was used for external calibration, with the objective of controlling the mass accuracy from 40 to 2000 *m/z* (<5 ppm) and mass resolution (>14,000 FWHM). The external calibration was performed prior to starting the analysis, followed by five consecutive injections of QC to reach the initial equilibrium. A QC was injected for every 6 wine samples in the randomized sequence, resulting in a total of 408 raw files acquired for each data set (175 in ESI+ and ESI− mode, with 29 QC). In the data acquisition, the rack temperature for vials was settled at 4 °C. The raw files obtained by UPLC-QToF MS analysis are available for download from the MetaboLights [51] public repository (https://www.ebi.ac.uk/metabolights/MTBLS2413), accessed on 30 November 2021 and tentatively release in February 2022.

The UPLC–MS/MS data acquisition was performed using the same conditions described above, but in V mode, using leucine enkephalin as lock mass and performing an external calibration with sodium formiate solution; the data were acquired in centroid mode with a scan duration of between 0.2 and 0.5 s. The source parameters for each mode (ESI+ and ESI−) were constant, but the transfer collision energy was in the range of 5 to 30 kV.

### 4.4. Data Analysis

For the untargeted analysis, the raw files were processed with Progenesis QI software (version 2.4, nonlinear Dynamics) for the principal component analysis (PCA) during the data acquisition, with the purpose of controlling the clustering of QC injections. The parameters for the data processing are as follows: software default mode for the alignment, maximum level for pick picking. By default, the Progenesis QI software considers a group of isotopic and adduct features coming from the same metabolite as a “compound” [9,17,23]. Putative biomarkers were considered the “compounds”/features that, according to analysis of variance (ANOVA), had a *p*-value ≤ 0.01 and maximum fold range of ≥2. The statistical analysis also considered the *q*-value, a maximum discovery rate (FDR)-adjusted *p*-value threshold of 0.01 for all the putative biomarkers.

The annotation was performed manually with a mass accuracy of 5 ppm, taking into account the isotopic pattern and in accordance with the four levels described by Sumner et al. [52]. An external database, as well as an internal wine metabolome database which includes more than 500 metabolites from different class chemical groups, were used for this purpose [9,12,16,17,23,24]. For a few compounds, a higher tolerance (less than 10 ppm) was accepted, due to an instrument limitation (intensity or *m/z* values registered for the compound as too high or too low) [24]. A semi manual integration of the annotated compounds (biomarkers and well-known wine metabolites) by the TargetLynx tool from MassLynx 4.1 software was performed. The parameters of the integration were 0.08 Da for the chromatogram mass window, 0.2 min for the retention time window, and smoothing iterations of 1 and width of 2. The integrated data were uploaded to the MetaboAnalyst online platform (https://www.metaboanalyst.ca), version 5.0 [53] (accessed on 18 February 2021). The data were treated for the heatmap plot without normalization, data transformation, and missing value estimation and with the Pareto scale. The visualization of the heat map was obtained by means of the Ward clustering algorithm, Euclidean distance, and using the group average option and is available in the Supplementary Information of this paper. Additionally, some figures and statistical analysis using the integrated data (correlation, one-way ANOVA, and the subsequent Tukey’s honestly significant difference test) were performed using R version 3.6.0 and R studio version 1.4.1717. The following packages were used for figures: “leaflet”, “viridis”, “ggplot2”, “magrittr”, “corrplot”,” moonBook”,” webr”, and “multcomp”.

The management of all data and metadata was made according to the FAIR guidelines for grapevine and wine studies [54].

## 5. Conclusions

The wine metabolomic fingerprint characterization makes it possible to gain new insight into the Chilean Cabernet Sauvignon chemical profile, according to a categorical company quality classification. The results highlighted the relevance of diverse chemical groups which characterized premium and standard wines. Small peptides are relevant to discriminate between quality groups. The standard wines had higher levels of stilbenoids and sulfonated compounds, while the premium wines were better characterized by polyphenols (flavanols, flavonols, anthocyanins) and phenolic acids. Some metabolites, such as flavan-3-ol hexoside and acetylated flavanols, were described for the first time in Chilean Cabernet Sauvignon wines. The results shed light on a possible sulfonation mechanism/reaction(s) not related to aging, and probably grape matrix-dependent. Further research is needed on Cabernet Sauvignon wines from other vintages to prove this hypothesis.

## Data Availability

Raw data can be download from the MetaboLights public repository (https://www.ebi.ac.uk/metabolights/MTBLS2413, accessed on 30 November 2021).

## Supplementary Materials

The following are available online at https://www.mdpi.com/article/10.3390/metabo11120829/s1, Figure S1: valley geographic distribution of wine samples, Figure S2: schematic representation of tryptophan catabolites, stilbenoids and cinnamic acids according to the valley, Figure S3: Correlation plot clustered between sulfonated metabolites and anthocyanidins-pigments compounds in premium and standard Cabernet Sauvignon wines, Figure S4: The Heatmap used for the Figure 3, Figure 4, Figure 5, Figure 6 and Figure 7 for quality groups, Table S1: Metadata information and basic enological analysis for the grape must and vineyards, Table S2: Metadata information and basic enological analysis for experimental wines, Table S3 and S4: biomarkers for the Premium and Standard Quality groups in LC–MS in ESI+ and ESI−, Tables S5 and S6: data analysis (*p*-value and *q*-value) and raw data integration for biomarkers, markers, and compounds with oenological relevance for sub-quality groups (P1, P2, S1 and S2), Tables S7–S10: Basic enological comparison between premium and standard wine groups and subgroups, Tables S11–S15: mean and standard deviation for semi-quantification metabolites from raw files by quality or valley.

## References

[1] International Organisation of Vine and Wine (OIV) Distribution of the World’s Grapevine Varieties. https://www.oiv.int/public/medias/5888/en-distribution-of-the-worlds-grapevine-varieties.pdf.

[2] Hernández A. (1997). Introducción al Vino de Chile.

[3] Anderson K., Nelgen S. (2020). Which Winegrape Varieties Are Grown Where? A Global Empirical Picture.

[4] Servicio Agrícola Ganadero (SAG) Catastro Vitícola Nacional. https://www.sag.gob.cl/noticias/sag-presenta-catastro-viticola-nacional-2019.

[5] Sarricolea P., Herrera-Ossandon M., Meseguer-Ruiz Ó. (2017). Climatic regionalisation of continental Chile. J. Maps.

[6] Montes C., Perez-Quezada J.-F., Peña-Neira A., Tonietto J. (2012). Climatic potential for viticulture in Central Chile. Aust. J. Grape Wine Res..

[7] Ribereau-Gayon P., Glories Y., Maujean A., Dubourdieu D. (2021). Handbook of Enology, Volume 2: The Chemistry of Wine Stabilization and Treatments.

[8] Cuadros-Inostroza A., Giavalisco P., Hummel J., Eckardt A., Willmitzer L., Peña-Cortés H. (2010). Discrimination of wine attributes by metabolome analysis. Anal. Chem..

[9] Arapitsas P., Ugliano M., Marangon M., Piombino P., Rolle L., Gerbi V., Versari A., Mattivi F. (2020). Use of untargeted liquid chromatography–mass spectrometry metabolome to discriminate Italian monovarietal red wines, produced in their different terroirs. J. Agric. Food Chem..

[10] Cuadros-Inostroza Á., Verdugo-Alegría C., Willmitzer L., Moreno-Simunovic Y., Vallarino J.G. (2020). Non-targeted metabolite profiles and sensory properties elucidate commonalities and differences of wines made with the same variety but different cultivar clones. Metabolites.

[11] Arapitsas P., Scholz M., Vrhovsek U., Di Blasi S., Biondi Bartolini A., Masuero D., Perenzoni D., Rigo A., Mattivi F. (2012). A metabolomic approach to the study of wine micro-oxygenation. PLoS ONE.

[12] Arapitsas P., Ugliano M., Perenzoni D., Angeli A., Pangrazzi P., Mattivi F. (2016). Wine metabolomics reveals new sulfonated products in bottled white wines, promoted by small amounts of oxygen. J. Chromatogr. A.

[13] Arapitsas P., Guella G., Mattivi F. (2018). The impact of SO2 on wine flavanols and indoles in relation to wine style and age. Sci. Rep..

[14] Ma L., Watrelot A.A., Addison B., Waterhouse A.L. (2018). Condensed tannin reacts with SO2 during wine aging, yielding flavan-3-ol sulfonates. J. Agric. Food Chem..

[15] Roullier-Gall C., David V., Hemmler D., Schmitt-Kopplin P., Alexandre H. (2020). Exploring yeast interactions through metabolic profiling. Sci. Rep..

[16] Arapitsas P., Speri G., Angeli A., Perenzoni D., Mattivi F. (2014). The influence of storage on the “chemical age” of red wines. Metabolomics.

[17] Ontañón I., Sánchez D., Sáez V., Mattivi F., Ferreira V., Arapitsas P. (2020). Liquid chromatography—Mass spectrometry-based metabolomics for understanding the compositional changes induced by oxidative or anoxic storage of red wines. J. Agric. Food Chem..

[18] Sáenz-Navajas M.P., Avizcuri J.M., Ballester J., Fernández-Zurbano P., Ferreira V., Peyron D., Valentin D. (2015). Sensory-active compounds influencing wine experts’ and consumers’ perception of red wine intrinsic quality. LWT—Food Sci. Technol..

[19] Gambetta J.M., Schmidtke L.M., Wang J., Cozzolino D., Bastian S.E., Jeffery D.W. (2017). Relating expert quality ratings of australian chardonnay wines to volatile composition and production method. Am. J. Enol. Vitic..

[20] Sherman E., Coe M., Grose C., Martin D., Greenwood D.R. (2020). Metabolomics approach to assess the relative contributions of the volatile and non-volatile composition to expert quality ratings of pinot noir wine quality. J. Agric. Food Chem..

[21] Serni E., Pedri U., Valls J., Sanoll C., Dordevic N., Überegger E., Robatscher P. (2020). Chemical description and organoleptic evaluation of Pinot noir wines from different parts of Italy: A three year investigation. ONEO One.

[22] Becerra-Rodríguez C., Marsit S., Galeote V. (2020). Diversity of oligopeptide transport in yeast and its impact on adaptation to winemaking conditions. Front. Genet..

[23] Moro L., Da Ros A., Vieira Da Mota R., Purgatto E., Mattivi F., Arapitsas P. (2020). LC-MS untargeted approach showed that methyl jasmonate application on Vitis labrusca L. grapes increases phenolics at subtropical Brazilian regions. Metabolomics.

[24] Shahaf N., Franceschi P., Arapitsas P., Rogachev I., Vrhovsek U., Wehrens R. (2013). Constructing a mass measurement error surface to improve automatic annotations in liquid chromatography/mass spectrometry based metabolomics. Rapid Commun. Mass Spectrom..

[25] Fabre S., Absalon C., Pinaud N., Venencie C., Teissedre P.L., Fouquet E., Pianet I. (2014). Isolation, characterization, and determination of a new compound in red wine. Anal. Bioanal. Chem..

[26] Favre G., González-Neves G., Piccardo D., Gómez-Alonso S., Pérez-Navarro J., Hermosín-Gutiérrez I. (2018). New acylated flavonols identified in *Vitis vinifera* grapes and wines. Food Res. Int..

[27] Delcambre A., Saucier C. (2012). Identification of new flavan-3-ol monoglycosides by UHPLC-ESI-Q-TOF in grapes and wine. J. Mass Spectrom..

[28] Bonaldo F., Guella G., Mattivi F., Catorci D., Arapitsas P. (2020). Kinetic investigations of sulfite addition to flavanols. Sci. Rep..

[29] Yamamoto S., Shiga K., Kodama Y., Imamura M., Uchida R., Obata A., Bamba T., Fukusaki E. (2014). Analysis of the correlation between dipeptides and taste differences among soy sauces by using metabolomics-based component profiling. J. Biosci. Bioeng..

[30] Zhao C.J., Schieber A., Gänzle M.G. (2016). Formation of taste-active amino acids, amino acid derivatives and peptides in food fermentations—A review. Food Res. Int..

[31] Zhou M., Bu T., Zheng J., Liu L., Yu S., Li S., Wu J. (2021). Peptides in brewed wines: Formation, structure, and function. J. Agric. Food Chem..

[32] Sun X., Udenigwe C.C. (2020). Chemistry and biofunctional significance of bioactive peptide interactions with food and gut components. J. Agric. Food Chem..

[33] Sáenz-Navajas M.P., Fernández-Zurbano P., Ferreira V. (2012). Contribution of nonvolatile composition to wine flavor. Food Rev. Int..

[34] Miyamura N., Iida Y., Kuroda M., Kato Y., Yamazaki J., Mizukoshi T., Miyano H. (2015). Determination and quantification of kokumi peptide, γ-glutamyl-valyl-glycine, in brewed alcoholic beverages. J. Biosci. Bioeng..

[35] Nikolantonaki M., Julien P., Coelho C., Roullier-Gall C., Ballester J., Schmitt-Kopplin P., Gougeon R.D. (2018). Impact of Glutathione on Wines Oxidative Stability: A Combined Sensory and Metabolomic Study. Front. Chem..

[36] Schneider V. (2014). Atypical aging defect: Sensory discrimination, viticultural causes, and enological consequences. A review. Am. J. Enol. Vitic..

[37] Marsit S., Sanchez I., Galeote V., Dequin S. (2016). Horizontally acquired oligopeptide transporters favour adaptation of *saccharomyces cerevisiae* wine yeast to oenological environment. Environ. Microbiol..

[38] Hellwig M. (2020). Analysis of protein oxidation in food and feed products. J. Agric. Food Chem..

[39] Gawel R., Schulkin A., McRea J., Hack J., Pearson W., Nandorfy D.E., Smith P. Beyond phenolic bitterness: Tryp-tophol-bisulfites identified as a potential new class of bitter compounds in white wine. Proceedings of the 17th Australian Wine Industry Technical Conference.

[40] Álvarez-Fernández M.A., Carafa I., Vrhovsek U., Arapitsas P. (2020). Modulating wine aromatic amino acid catabolites by using *Torulaspora delbrueckii* in sequentially inoculated fermentations or *Saccharomyces cerevisiae* alone. Microorganisms.

[41] Piombino P., Pittari E., Gambuti A., Curioni A., Giacosa S., Mattivi F., Parpinello G., Rolle L., Ugliano M., Moio L. (2020). Preliminary sensory characterisation of the diverse astringency of single cultivar Italian red wines and correlation of sub-qualities with chemical composition. Aust. J. Grape Wine Res..

[42] García-Estévez I., Ramos-Pineda A., Escribano-Bailón M.T. (2018). Interactions between wine phenolic compounds and human saliva in astringency perception. Food Funct..

[43] Renaud S., De Lorgeril M. (1992). Wine, alcohol, platelets, and the French paradox for coronary heart disease. Lancet.

[44] Flamini R., De Rosso M. (2019). High-resolution mass spectrometry and biological properties of grapevine and wine stilbenoids. Studies in Natural Products Chemistry.

[45] Gaudette N.J., Pickering G.J. (2011). Sensory and chemical characteristics of *trans*-resveratrol-fortified wine. Aust. J. Grape Wine Res..

[46] Poklar Ulrih N., Opara R., Korošec M., Wondra M., Abram V. (2020). Part II. Influence of *Trans*-resveratrol addition on the sensory properties of ‘Blaufränkisch’ red wine. Food Chem. Toxicol..

[47] Ferrero-del-Teso S., Suárez A., Jeffery D.W., Ferreira V., Fernández-Zurbano P., Sáenz-Navajas M.P. (2020). Sensory variability associated with anthocyanic and tannic fractions isolated from red wines. Food Res. Int..

[48] Ferrer-Gallego R., Soares S., Mateus N., Rivas-Gonzalo J., Escribano-Bailon M.T., Freitas V. (2015). New anthocyanin—Human salivary protein complexes. Langmuir.

[49] Schober D., Legues M., Guidez H., Caris Maldonado J.C., Vargas S., Gonzalez Rojas A. How geographical origin and vineyard management influence cv. Cabernet-Sauvignon in Chile—Machine learning based quality prediction. Proceedings of the XIIIth International Terroir Congress.

[50] Arapitsas P., Mattivi F. (2018). LC-MS untargeted protocol for the analysis of wine. Metabolic Profiling.

[51] Haug K., Cochrane K., Nainala V.C., Williams M., Chang J., Jayaseelan K.V., O’Donovan C. (2019). MetaboLights: A resource evolving in response to the needs of its scientific community. Nucleic Acids Res..

[52] Sumner L.W., Amberg A., Barrett D., Beale M.H., Beger R., Daykin C.A., Fan T.W.M., Fiehn O., Goodacre R., Griffin J.L. (2007). Proposed minimum reporting standards for chemical analysis. Chemical analysis working group (CAWG) metabolomics standards initiative (MSI). Metabolomics.

[53] Pang Z., Chong J., Zhou G., Anderson D., Morais D.L., Chang L., Barrette M., Gauthier C., Jacques P., Li S. (2021). MetaboAnalyst 5.0: Narrowing the gap between raw spectra and functional insights. Nucleic Acids Res..

[54] Savoi S., Arapitsas P., Duchêne É., Nikolantonaki M., Ontañón I., Carlin S., Schwander F., Gougeon R.D., Silva Ferreira A., Theodoridis G. (2021). Grapevine and Wine Metabolomics-Based Guidelines for FAIR Data and Metadata Management. Metabolites.

