# Supplementary material for: LC–MS-Based Metabolomics Discriminates Premium from Standard Chilean cv. Cabernet Sauvignon Wines from Different Valleys
Source: Metabolites. 2021 Nov 30;11(12):829. doi: 10.3390/metabo11120829 (PMC8707972; doi:10.3390/metabo11120829)
Supplement: Supplementary file 1 [file metabolites-11-00829-s001.zip › Supplementary files/suplementary material.pdf]

LC–MS-Based Metabolomics Discriminates Premium from Standard Chilean cv. Cabernet Sauvignon Wines from Different Valleys

Vania Sáez[1], Doreen Schober[2], Álvaro González[2], Panagiotis Arapitsas[1,3 *].

[1]Food Quality and Nutrition Department, Research and Innovation Centre, Fondazione Edmund Mach, Via Edmund Mach 1, 38010 San Michele All'Adige, Italy.

[2]Center for Research and Innovation, Viña Concha y Toro, Km 10 Ruta K-650, 3550000 Pencahue, Región del Maule, Chile.

[3]Department of Wine, Vine and Beverage Sciences, School of Food Science, University of West Attica, Ag. Spyridonos str, Egaleo, 12243 Athens, Greece.

Supporting Information

Supplementary Tables

Table S1. Metadata information and basic enological analysis for the grape must and vineyards. *(Separated file)*

Table S2: Metadata information and basic enological analysis for experimental wines. *(Separated file)*

Table S3. ESI+ features with *p*-value≤0.01, *q*-value≤0.05 and max folder >2. *(Separated file)*

Table S4. ESI- features with *p*-value≤0.01, *q*-value≤0.05 and max folder >2. *(Separated file)*

Table S5. Tentative annotated metabolites of oenological interest. *(Separated file)*

Table S6. Integration (expressed in area) of the tentative annotated biomarkers. *(Separated file)*

Table S7: Enological parameters measured in grape must according to Premium (P) and Standard (S) quality groups.

Table S8: Enological parameters in grape must according to subquality groups.

Table S9: Enological parameters of experimental Cabernet Sauvignon wines according to Premium (P) and Standard (S) quality groups.

Table S10: Enological parameters of experimental Cabernet Sauvignon wines according to subquality groups.

Table S11: Integration means from raw files for amino acids, peptides, and non-flavonoids according to subquality groups.

Table S12: Integrated areas means and standard deviation from raw files for tryptophan catabolites, stilbenoids and cinnamic acids for analyzed wines according to the valley.

Table S13: Integrated areas means and standard deviation from raw files for the flavonoids and the anthocyanins of Figure 5-6.

Table S14: Integrated areas means and standard deviation from raw files for the flavonols of Figure 7A.

Table S15: Integrated areas means and standard deviation from raw files for metabolites of Figure 7B.

Supplementary Figures

Figure S1. valley geographic distribution of wine samples.

Figure S2. schematic representation of tryptophan catabolites, stilbenoids and cinnamic acids according to the valley. **Li:**Limarí, **Val:** valparaíso (Aconcagua valley), **Cur:** Curicó, **Mau:** Maule, **Ca:** Cachapoal, **Col:**Colchagua, **It:**Itata and **Mai:** Maipo.

Figure S3. Correlation plot clustered between sulfonated metabolites and anthocyanidins-pigments compounds in premium and standard Cabernet Sauvignon wines (α=0.05).

Figure S4. The Heatmap used for the Figures F3-F7 for P1, P2, S1 and S2 quality groups. *(Separated file)*

Supplementary Table S7: Enological parameters measured in grape must according to Premium (P) and Standard (S) quality groups.

Parameter	P	S	*p*-value
**Brix°**	24.1±2.5	22.7±1,4	0.02
**Ammonium (g/L)**	82±50.3	85.8±29.7	0.74
**PAN (g/L)**	55.4±29.5	61.8±20.8	0.39
**YAN(g/L)**	148.8±65.9	147.7±47.0	0.97

Supplementary Table S8: Enological parameters in grape must according to subquality groups.

Parameter	P1	P2	S1	S2	*p*-value
**Brix°**	25.07±1.16[a]	23.66±2.8[a]	22.52±1.53[b]	22.92±1.38[a]	≤0.05
**Ammonium (g/L)**	104.31±79.36	73.32±32.62	92.09±30.90	76.46±26.50	≥0.05
**PAN (g/L)**	65.18±31.72	51.66±28.63	64.44±15.60	57.71±27.25	≥0.05
**YAN(g/L)**	195.76±76.95[a]	130.02±52.62[b]	156.79±44.10[a]	134.15±50.15[a]	≤0.05

Letters indicated pairwise comparison with post-hoc Tukey test after anova one-way. Different superscript letters indicated statistically difference (*p*-value≤0.005) between P1, P2, S1 and S2 groups.

Supplementary Table S9: Enological parameters of experimental Cabernet Sauvignon wines according to Premium (P) and Standard (S) quality groups.

Parameter	P	S	*p-value*
**pH**	3.45±0.04	3.46±0.03	0.24
**TSO2 (ppm)**	45.2±5.06	47.4±5.66	0.16
**F SO2 (ppm)**	38.3±4.82	41.7±5.34	0.02
**A (% v/v)**	13.3 ±0.89	13.3±0.88	0.30

Supplementary Table S10: Enological parameters of experimental Cabernet Sauvignon wines according to subquality groups.

Parameter	P1	P2	S1	S2	*p-value*
**pH**	3.45±0.04	3.44±0.04	3.45±0.03	3.46±0.03	≥0.05
**TSO2 (ppm)**	43.42±4.3	45.93±5.26	47.83±6.39	46.72±4.59	≥0.05
**F SO2 (ppm)**	37.21±3.88	38.68±5.17	41.47±6.28	41.92±3.79	≥0.05
**A (% v/v)**	13.55±0.22[a,b]	13.79±0.52[a]	12.70±0.94[b]	13.07±0.84[a,b]	≤0.05

Letters indicated pairwise comparison with post-hoc Tukey test after one-way ANOVA. Different superscript letters indicated statistically difference (*p*-value≤0.005) between P1, P2, S1 and S2 groups.

Supplementary Table S11: Integration means from raw files for amino acids, peptides, and non-flavonoids according to subquality groups.

compound	P1	P2	S1	S2
**valine**	13.09±2.60^{b}	14.59±3.27^{a,b}	19.04±6.73^{a}	16.96±3.40^{a}
**arginine**	14.19±6.98^{a,b}	8.21±3.51^{b}	17.74±10.35^{a}	9.28±7.95^{b}
**leucine**	3.08±1.11^{a}	2.18±0.62^{b}	2.37±1.53^{b}	1.80±0.80^{b}
**isoleucine**	0.35±0.15^{a,b}	0.26±0.09^{b}	0.38±0.28^{a}	0.24±0.12^{b}
**proline**	168.82±14.22^{a}	144.38±25.11^{b}	141.90±21.94^{b}	121.74±30.16^{c}
**tyrosine**	9.58±2.17^{a}	7.32±1.12^{b}	9.18±3.93^{a}	7.06±1.50^{b}
**tryptophan**	1.32±0.59^{a}	0.79±0.31^{b}	1.16±1.02^{a}	0.59±0.28^{b}
**phenylalanine**	9.60±3.42^{a}	6.61±1.74^{b}	8.51±5.65^{a}	5.87±2.67^{b}
**trytophol 2-sulfate**	2.79± 0.77^{b}	3.73±1.18^{a,b}	4.35±1.76^{a}	4.90±1.79^{a}
**indole 3 lactic-acid**	28.44±8.67^{b}	34.36±22.97^{b}	62.13±51.22^{a}	40.13±18.04^{b}
**indole lactic acid glucoside**	1667.22±95.47	1601.63±165.29	1645.20±290.03	1590.56±320.95
**indole lactic glucoside sulfonate**	13.39±4.08^{b}	18.16±4.69^{b}	21.99±9.89^{a}	22.40±6.84^{a}
**beta-glucogallin**	19.05±3.80^{a}	14.34±3.57^{b}	13.62±8.52^{b}	12.37±4.10^{b}
**galloyl hexoside**	7.69±6.58^{a}	6.68±6.12^{a}	1.20±2.04^{b}	1.78±2.27^{b}
**Cysteine sulfonate**	1.19±0.23	1.81±2.63	1.61±0.78	1.30±0.63
**3,4-hydroxybenzoic acid**	1.33±0.36^{a}	1.10±0.42^{a}	0.79±0.55^{b}	0.82±0.40^{b}
**4-hydroxybenzoic acid**	5.54±1.58^{a}	4.36±0.99^{b}	3.55±1.02^{b}	3.85±1.00^{b}
***trans*-fertaric acid**	12.22±2.06^{a}	9.94±1.80^{b}	8.76±1.52^{c}	10.05±1.49^{b}
***O*-coumaric acid**	2.20±0.63^{b}	3.20±1.23^{a}	3.87±1.54^{a}	3.44±1.61^{a}
**caftaric acid**	8.70±1.84^{a}	8.08±1.75^{a,b,c}	7.36±1.83^{c}	9.37±2.25^{a}
***trans*-coutaric acid**	49.40±11.79^{a,b}	44.13±7.76^{b,c}	40.41±8.83^{c}	50.29±10.74^{a}
**caffeic.acid**	29.93±7.39^{c}	35.45±9.29^{b}	42.10±11.29a	39.88±13.52^{a,b}
***cis*-coutaric acid**	3.37±0.49	3.42±0.73	3.19±0.54	3.46±0.46
**Leu-leu-leu**	1.51±0.81	0.92±0.41	1.44±1.55	0.79±0.51
**Ethyl-caffeate**	67.44±13.20^{b}	89.97±27.39^{a}	98.06±30.54^{a}	101.98±42.05^{a}
**vanillic acid**	5.69±0.93^{a}	4.68±0.89^{b}	3.76±1.23^{c}	4.03±0.83^{c}
***trans*-resveratrol**	5.89±2.13^{c}	7.97±3.96^{b}	9.32±3.66^{b}	13.94±6.42^{a,b}
***trans*-piceid**	22.46±10.35^{c}	25.29±13.28^{b,c}	27.59±20.12^{b}	39.34±25.70^{a}
***trans*-piceatannol**	3.61±1.28^{b}	4.34±2.52^{b}	4.18±1.54^{b}	5.50±2.51^{a}
**pallidol**	1.97±0.98^{b}	2.22±2.36^{b}	2.71±2.74^{b}	5.05±4.34^{a}
**ferulic acid-*O*-glucoside**	2.00±0.56^{a}	1.63±0.57^{b}	1.54±0.55^{b}	1.63±0.40^{b}
**gallic acid**	54.62±11.75^{a}	46.00±6.89^{b}	44.33±8.07^{b}	42.18±6.08^{b}
**egallic acid**	95.52±28.06^{a}	73.28±21.23^{b}	62.50±19.26^{b}	75.19±40.43^{b}
**α-aminoadipic acid**	1.82±0.20^{a}	1.73±0.268^{a,b}	1.61±0.27^{b,c}	1.53±0.32^{c}
**glutathione**	2.21±0.38^{a}	1.23±0.44^{b}	1.09±0.39^{b}	1.21±0.89^{b}
**gluthathione S-Sulfonate**	22.46±4.35^{a}	23.99±18.37^{a}	15.03±10.73^{a,b}	14.82±7.07^{b}
**tyr-ala**	1.60±0.78^{a}	1.05±0.41^{b}	1.59±1.33^{a}	0.94±0.44^{b}
**pro-thr**	2.37±0.75	1.98±0.45	2.48±1.56	2.07±0.62

**t 4-hydroxy proline**	0.26±0.08	0.26±0.09	0.29±0.08	0.24±0.08
**gly-his**	0.17±0.10^{b}	0.13±0.08^{b}	0.30±0.33^{a}	0.10±0.06^{b}

Letters indicated pairwise comparison with HSD Tukey test after one-way ANOVA. Different superscript letters indicated statistically difference (p-value≤0.005) between P1, P2, S1 and S2 groups.

Supplementary Table S12: Integrated areas means and standard deviation from raw files for tryptophan catabolites, stilbenoids and cinnamic acids for analyzed wines according to the valley.

compound	Cachapoal	Colchagua	Curicó	Itata	Limarí	Maipo	Maule	Aconcagua
**tryptophan**	0.57±0.25[b]	0.87±0.36[a]	1.25±1.49[a]	1.13±0.23[a]	0.70±0.34[b]	1.39±0.52[a]	0.77±0.26[b]	0.64±0.11[b]
**indole 3-lactic acid**	39.58±21.67	31.48±14.47	41.96±13.18	20.87±0.89	53.72±15.71	36.90±26.48	55.78±54.36	44.52±0.49
**indole lactic glucoside**	1562.97±187.71[b]	1672.11±190.71[b]	1677.81±198.81[b]	1850.43±44.77[a]	2002.54±291.35[a]	1693.27±101.41[b]	1469.67±254.00[b]	1793.31±58.40[a,b]
**indole lactic glucoside sulfonate**	18.11±5.36[b]	19.81±4.81[a,b]	20.87±4.71[a,b]	16.91±1.95[b]	29.13±3.63[a]	14.71±5.49[b]	20.37±10.56[a,b]	23.23±2.38[a,b]
**tryptophol sulfonate**	4.72±2.34[a]	3.83±1.31[a]	4.10±0.87[a]	4.05±0.60[a]	4.65±0.84[a]	2.86±0.81[b]	4.32±1.85[a]	4.56±1.13[a]
**trans-resveratrol**	8.50±3.27[a,b]	6.54±3.75[b]	10.27±3.07[a]	10.78±0.46[a]	11.82±4.47[a]	6.29±1.71[b]	12.21±6.46[a]	7.78±0.17[a,b]
**trans-piceid**	25.89±9.52	26.83±18.28	36.13±21.37	21.48±1.71	26.62±2.95	22.54±9.94	31.46±24.93	16.27±1.97
**trans-piceatannol**	4.30±1.86[a]	3.74±2.22[b]	4.17±0.85[a]	3.53±0.27[b]	5.10±1.23[a]	3.50±1.27[b]	5.53±2.82[a]	3.95±0.13[a,b]
**pallidol**	1.81±1.13	3.25±3.75	3.51±2.65	0.88±0.11	3.09±1.23	1.96±0.89	3.56±3.92	0.82±0.09
**3,4-hydroxybenzoic acid**	1.04±0.49	1.14±0.50	0.93±0.47	1.18±0.10	0.53±0.22	1.12±0.44	0.84±0.50	1.06±0.09
**4-hydroxybenzoic acid**	4.07±1.03[b]	3.99±0.83[b]	3.44±0.95[b]	5.48±0.08[a]	3.94±0.94[b]	5.27±1.68[a]	4.06±1.22[b]	4.25±0.35[a]

Letters indicated pairwise comparison with HSD Tukey test after one-way ANOVA. Different superscript letters indicated statistically difference (*p*-value≤0.005) between different origin valley.

Supplementary Table S13: Integrated areas means and standard deviation from raw files for the flavonoids and the anthocyanins of Figure 5-6.

compound	P1	P2	S1	S2
**naringenin**	13.44±2.98	15.32±5.63	13.32±5.15	15.32±4.02
**luteolin**	2.92±0.70[a]	2.39±0.55[b]	2.01±0.65[b]	2.48±0.80[b]
**luteolin 7-glucoside**	35.81±15.81[a]	16.48±17.81[b]	4.93±7.90[c]	13.10±12.63[b,c]
**quercetin**	553.09±102.89[a]	504.30±177.90[a]	358.62±238.32[b]	502.01±235.36[a]
**quercetin 3-glucoside**	607.56±266.15[a]	249.08±257.74[b]	75.35±122.32[c]	202.75±181.31[b,c]
**quercetin 3-glucuronide**	608.52±127.66[a]	418.62±160.01[b]	310.98±120.95[c]	405.53±189.08[b]
**quercetin 4-glucoside**	157.32±61.02[a]	84.72±68.05[b]	50.82±48.36[c]	110.31±81.79[b]
**quercetin -xyloside**	4.40±2.72[a]	2.51±3.04[b]	1.35±1.92[b]	2.53±2.02[b]
**isorhamnetin**	115.71±23.47[a]	127.10±71.67[a]	86.78±65.68[b]	115.53±71.75[a]
**isorhamnetin 3-glucoside**	168.90±61.70[a]	93.63±63.22[b]	37.66±34.70[c]	77.64±44.68[b]
**isorhamnetin 3-(6"-acetyl)-glucoside**	27.83±5.70[a]	20.04±6.03[b]	15.32±4.92[c]	16.60±5.69[c]
**cyanidin 3-glucoside**	43.21±28.48[a]	37.62±19.83[a]	24.90±12.12[b]	33.95±23.03[a,b]
**cyanidin 3-(6"-acetyl)-glucoside**	35.14±21.18[a]	29.52±15.33[a]	18.94±10.43[b]	27.28±17.63[a]
**cyanidin 3-(6"-p-coumaroyl)-glucoside**	11.10±6.78[a]	9.76±3.61[a]	7.31±3.36[b]	10.58±8.47[a,b]
**peonidin 3-glucoside**	410.28±132.24[a]	365.63±95.94[a]	309.70±103.09[b]	337.55±125.94[a,b]
**peonidin3-(6"-acetyl)-glucoside**	287.05±71.35[a]	254.66±55.61[a]	211.38±66.11[b]	236.76±76.99[b]
**peonidin3-(6"-p-coumaroyl)-glucoside**	131.60±41.16	121.34±35.23	108.34±40.82	122.52±66.45
**dihydrokaempferol**	7.058±1.68	7.14±1.62	6.61±1.99	6.88±1.10
**dihydroquercetin rhamnoside**	64.33±23.04[a]	48.42±16.73[b]	42.22±16.98[b]	51.51±32.74[a,b]
**kaempferol**	164.30±72.93[a]	109.92±81.98[a]	37.44±51.94[b]	108.25±127.33[a]
**kaempferol 3-glucoside**	92.46±66.04[a]	35.63±39.75[b]	14.86±13.58[c]	29.91±21.20[b,c]
**kaempferol 3-glucuronide**	13.87±3.23[a]	9.37±4.36[b]	6.58±3.46[c]	9.09±4.59[b]
**myricetin**	200.23±36.04	233.01±49.50	245.40±86.21	240.71±94.00
**myricetin 3-glucoside**	843.23±193.25[a]	516.90±213.39[b]	283.53±188.23[c]	463.82±251.21[b]
**myricetin 3-rhamnoside**	3.94±1.59	4.00±2.35	3.916±2.94	3.27±2.12
**laricitrin**	10.84±2.14	15.66±10.81	18.60±15.21	17.47±16.69
**laricitrin 3-(6"-acetyl)-glucoside**	1.69±0.67[a]	1.43±0.80[a]	1.02±0.68[b]	1.22±0.72[a]
**syringetin**	16.57±4.49[a]	13.34±2.73[b]	13.75±4.736[b]	13.36±3.49[a,b]
**syringetin 3-glucoside**	234.64±35.48[a]	192.79±43.60[b]	173.72±38.66[b]	170.99±39.48[b]
**syringetin 3-(6"-acetyl)-glucoside**	1.15±0.34[b]	1.22±0.51b	2.44±1.63[a]	1.04±0.68[c]
**delphinidin 3-glucoside**	654.00±224.65	680.83±232.79	570.56±252.80	690.12±335.34
**delphinidin 3-(6"-p-coumaroyl)-glucoside**	45.12±17.33	42.28±12.92	38.43±17.12	54.44±43.05
**delphinidin 3-(6"-acetyl)-glucoside**	277.74±90.69[a]	264.31±99.840[a]	206.36±101.63[b]	269.35±130.54[a,b]
**delphinidin 3,5-diglucoside**	39.15±15.93[a,b]	50.70±25.85[a]	20.82±17.80[c]	36.51±20.45[b]
**petunidin 3-glucoside**	960.07±230.30[a,b,c]	959.88±258.62[b]	775.00±286.67[c]	973.86±444.31[a]
**petunidin 3-(6"-p-coumaroyl)-glucoside**	92.33±27.89	81.89±23.56	69.95±27.67	94.36±67.79
**petunidin 3-(6"-acetyl)-glucoside**	448.14±116.62[a]	408.13±125.98[a]	320.03±133.33[b]	417.52±196.08[a]
**malvidin 3-glucoside**	7724.31±868.99	7598.47±852.24	7710.51±1436.24	7617.66±1553.8
**malvidin 3-(6"-acetyl)-glucoside**	4911.33±669.09	4448.10±575.81	4395.39±868.68	4429.66±979.11

**malvidin 3-(6"-p-coumaroyl)-glucoside**	1111.71±206.67	988.98±198.20	1013.63±270.54	1062.49±408.33
**catechin/epicatechin ethyl malvidin 3-glucoside**	28.32±17.66^{a}	14.54±8.34^{b}	10.27±5.90^{c}	10.24±7.18^{c}
**catechin/epicatechin ethyl malvidin 3-glucoside**	11.10±6.91^{a}	5.38±5.90^{b}	3.19±1.51^{c}	3.16±2.35^{c}
**catechin/epicatechin malvidin 3-glucoside**	116.86±24.27^{a}	111.28±15.16^{a}	100.40±25.70^{b}	119.69±37.38^{c}
**catechin/epicatechin-ethyl-malvidin 3-(6''-p-coumaroyl)-glucoside**	13.77±8.35^{a}	6.40±2.85^{b}	5.18±1.75^{b}	5.64±3.59^{b}
**malvidin 3-glucoside-4vinylphenol**	20.84±6.73	21.66±10.75	22.51±10.87	30.56±29.02
**pyranomalvidin 3-(6”-acetyl)-glucoside**	126.10±63.84^{a}	52.63±36.77^{b}	48.19±24.16^{b}	23.79±18.51^{b}
**pyranomalvidin 3-(6”-p-coumaroyl)-glucoside**	42.99±25.39^{a}	16.38±13.049^{b}	15.03±9.28^{b}	9.06±9.40^{b}
**carboxypyranomalvidin 3-(6”-acetyl)-glucoside**	168.96±29.47^{a}	108.20±32.42^{b}	101.04±32.84^{b}	88.00±30.30^{b}
**carboxypyranomalvidin 3-(6”-p-coumaroyl)-glucoside**	56.22±13.22^{a}	35.23±32.42^{b}	34.67±10.61^{b}	34.59±17.05^{b}
**methyl-pyrano-malvidin 3-glucoside**	73.21±17.78^{a}	48.49±18.99^{b}	33.26±13.22^{c}	28.73±10.29^{c}
**Pinotin**	88.52±17.24	82.51±23.71	71.63±26.35	101.04±84.05
**Pinotin A**	86.07±13.91^{a,b}	82.40±23.50^{a,b}	71.81±26.35^{b}	100.56±83.62^{a}
**Oxovitisin A**	10.51±3.57^{a}	5.77±2.66^{b}	5.11±1.50^{c}	3.73±1.71^{c}

Letters indicated pairwise comparison with HSD Tukey test after one-way ANOVA. Different superscript letters indicated statistically difference (p-value≤0.005) between P1, P2, S1 and S2.

Supplementary Table S14: Integrated areas means and standard deviation from raw files for the flavonols of Figure 7A.

compound	P1	P2	S1	S2
**Epicatechin**	146.44 ± 49.27^{a}	121.63 ± 35.23^{b}	$110.68\pm29.87^{b,c}$	95.683 ± 18.83^{c}
**Catechin**	232.58 ± 68.80^{a}	178.00 ± 39.95^{b}	$161.50\pm32.65^{b,c}$	152.37 ± 20.94^{c}
**PB3**	38.45 ± 15.01^{a}	29.34 ± 7.79^{b}	28.16 ± 9.37^{b}	23.134 ± 2.62^{b}
**PB4**	26.58 ± 9.63^{a}	20.49 ± 4.91^{b}	18.37 ± 4.82^{b}	18.917 ± 4.18^{b}
**PB2**	190.04 ± 39.32^{a}	159.53 ± 30.02^{b}	158.41 ± 24.05^{b}	156.488 ± 14.77^{b}
**caffeic acid-catechin condesation product**	0.32 ± 0.29^{a}	0.26 ± 0.18^{a}	0.21 ± 0.18^{b}	0.439 ± 0.43^{a}
**Proanthocyanidin trimer**	3.58 ± 1.25^{a}	2.65 ± 0.96^{b}	2.61 ± 1.04^{b}	3.167 ± 1.18^{b}
**Proanthocyanidin trimer**	1.93 ± 0.56^{a}	1.36 ± 0.56^{b}	1.38 ± 0.59^{b}	1.658 ± 0.55^{b}
**Proanthocyanidin trimer**	5.90 ± 2.19^{a}	4.41 ± 1.14^{b}	4.09 ± 1.30^{b}	5.151 ± 1.14^{b}
**Proanthocyanidin trimer**	3.57 ± 1.58^{a}	2.70 ± 0.80^{b}	2.50 ± 0.61^{b}	3.313 ± 0.89^{b}
**Proanthocyanidin trimer**	2.94 ± 1.61^{a}	1.79 ± 0.77^{b}	1.76 ± 0.66^{c}	2.029 ± 0.48^{b}
**Proanthocyanidin dimer**	58.94 ± 14.07^{a}	47.16 ± 8.51^{b}	47.67 ± 7.60^{b}	53.211 ± 7.66^{b}
**Proanthocyanidin dimer**	44.38 ± 11.32^{a}	36.54 ± 7.94^{b}	39.91 ± 8.02^{b}	41.153 ± 7.82^{b}
**Proanthocyanidin dimer**	6.34 ± 2.29^{a}	5.10 ± 1.12^{b}	4.64 ± 0.73^{c}	4.700 ± 0.72^{c}
**Proanthocyanidin dimer**	10.42 ± 2.95^{a}	7.49 ± 1.58^{b}	8.28 ± 1.75^{b}	8.810 ± 2.32^{b}
**flavan-3-ol hexoside**	3.47 ± 1.76^{a}	2.81 ± 1.48^{a}	1.62 ± 1.24^{b}	1.472 ± 0.64^{b}
**flavan-3-ol hexoside**	12.72 ± 4.74^{a}	11.35 ± 5.02^{a}	8.47 ± 3.41^{b}	7.070 ± 2.70^{b}
**epicatechin sulfonate**	10.43 ± 2.67^{b}	$12.63\pm3.19^{a,b}$	11.55 ± 2.88^{b}	15.184 ± 4.48^{a}
**procyanidin B2-4β-sulfonate**	0.47 ± 0.23^{b}	0.61 ± 0.30^{a}	$0.49\pm0.23^{a,b}$	0.809 ± 0.42^{a}
**prodelphinidin-4β-sulfonate**	1.88 ± 0.53^{b}	2.34 ± 1.02^{b}	2.75 ± 1.17^{b}	3.626 ± 1.99^{a}
**prodelphinidin-4β-sulfonate**	2.18 ± 0.41^{b}	3.55 ± 1.16^{b}	3.26 ± 1.45^{b}	4.575 ± 1.54^{a}

Letters indicated pairwise comparison with HSD Tukey test after one-way ANOVA. Different superscript letters indicated statistically difference (p-value≤0.005) between P1, P2, S1 and S2.

Supplementary Table S15: Integrated areas means and standard deviation from raw files for metabolites of Figure 7B.

compound	P1	P2	S1	S2
**D-pantothenic acid**	2.58 ± 0.48^{a}	2.03 ± 0.38^{b}	1.81 ± 0.54^{b}	1.40 ± 0.40^{c}
**uridine monophosphate**	18.20 ± 5.93^{a}	16.05 ± 4.01^{a}	6.76 ± 3.86^{c}	13.36 ± 4.96^{b}
**hypoxanthine**	$18.74\pm2.60^{a,b}$	15.21 ± 3.34^{b}	17.84 ± 5.67^{a}	$17.58\pm2.48^{a,b}$
**xanthine**	74.41 ± 5.95^{b}	$80.11\pm5.74^{a,b}$	72.03 ± 13.93^{b}	82.91 ± 7.42^{a}
**uridine or pseudouridine**	$7.84\pm2.31^{a,b}$	5.91 ± 1.21^{b}	9.70 ± 5.40^{a}	5.52 ± 1.15^{b}
**tartaric acid**	289.33±11.57	287.04±17.51	283.83±7.86	281.97±14.60
**riboflavina**	12.25 ± 1.27^{a}	9.80 ± 1.29^{b}	11.48 ± 2.66^{a}	9.30 ± 1.85^{b}
**choline**	121.77 ± 19.63^{b}	$133.33\pm17.75^{a,b}$	146.63 ± 45.81^{a}	$129.62\pm14.10^{a,b}$
**pantotheine sulfonate**	6.93±2.46	7.58±5.69	7.73±2.80	6.42±2.21
**malic acid**	4.99±0.63b	$5.78\pm1.26^{a,b}$	5.79 ± 1.15^{b}	5.95 ± 0.94^{a}
**Uracil**	2.07 ± 0.28^{b}	2.23 ± 0.39^{b}	$2.46\pm0.51^{a,b}$	2.89 ± 0.78^{a}

Letters indicated pairwise comparison with HSD Tukey test after one-way ANOVA. Different superscript letters indicated statistically difference (p-value≤0.005) between P1, P2, S1 and S2.

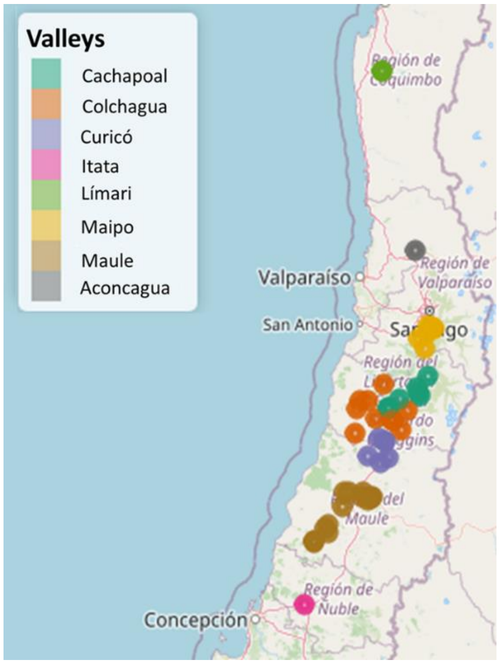

Supplementary Figure S1: valley geographic distribution of wine samples.

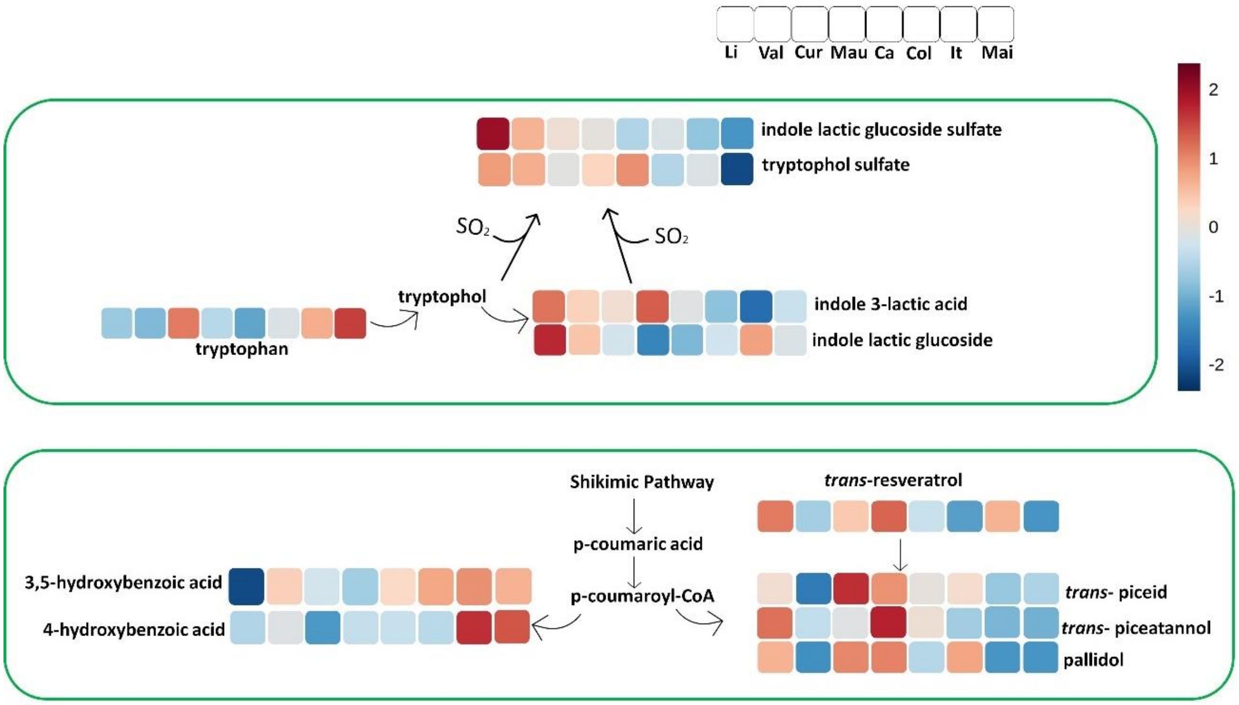

Supplementary Figure S2: schematic representation of tryptophan catabolites, stilbenoids and cinnamic acids according to the valley. **Li:**Limarí, **Val:** valparaíso (Aconcagua valley), **Cur:** Curicó, **Mau:** Maule, **Ca:** Cachapoal, **Col:**Colchagua, **It:**Itata and **Mai:** Maipo.

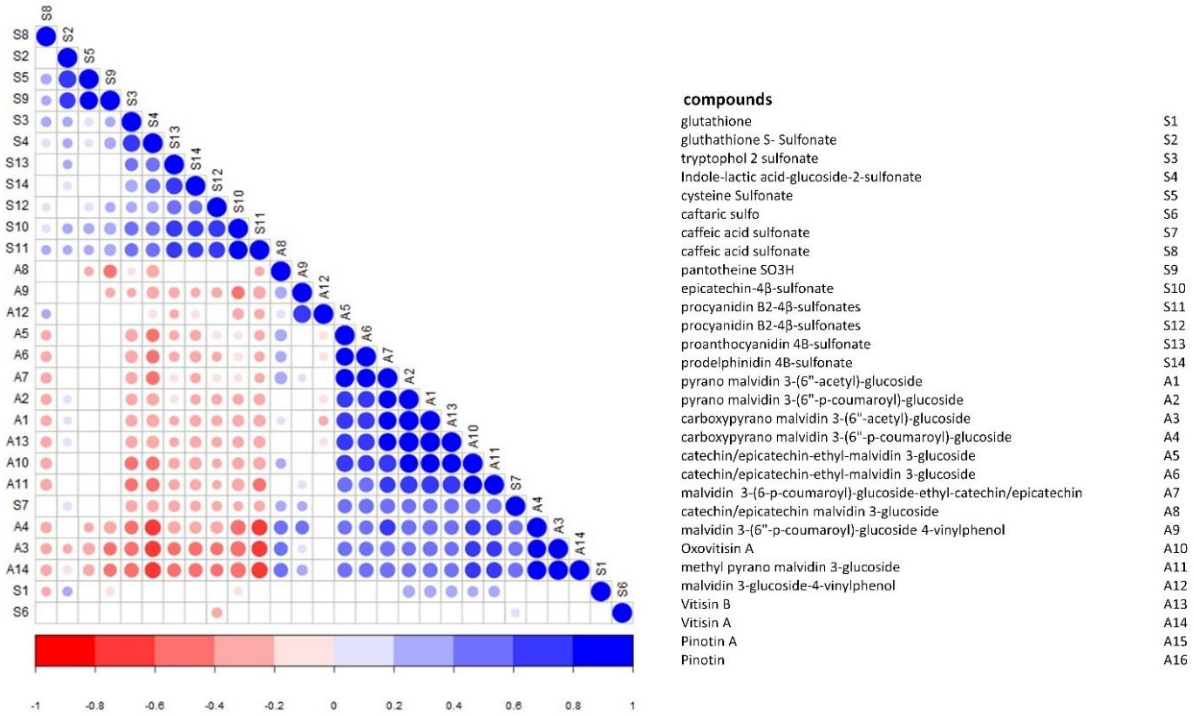

Supplementary Figure S3: Correlation plot clustered between sulfonated metabolites and anthocyanidins-pigments compounds in premium and standard Cabernet Sauvignon wines (α=0.05).

Safety Statement

This study does not include any new or unexpected significant hazards or risks associated material. A Table with all the chemicals and their Classification/Hazardous Identification can be found below.

Material Name	Material CAS-No	Classification according to Regulation (EC) No 1272/2008
Methanol	67-56-1	Flammable liquids (Category 2), H225 Acute toxicity, Oral (Category 3), H301 Acute toxicity, Inhalation (Category 3), H331 Acute toxicity, Dermal (Category 3), H311 Specific target organ toxicity - single exposure (Category 1), Eyes, Central nervous system, H370
Acetonitrile	75-05-8	Flammable liquids (Category 2), H225 Acute toxicity, Oral (Category 4), H302 Acute toxicity, Inhalation (Category 4), H332 Acute toxicity, Dermal (Category 4), H312 Eye irritation (Category 2), H319
Formic acid	64-18-6	Flammable liquids (Category 3), H226 Acute toxicity, Oral (Category 4), H302 Acute toxicity, Inhalation (Category 3), H331 Skin corrosion (Sub-category 1A), H314 Serious eye damage (Category 1), H318
2-Propanol	67-63-0	Flammable liquids (Category 2), H225 Eye irritation (Category 2), H319 Specific target organ toxicity - single exposure (Category 3), Central nervous system, H336
Leucine Enkephalin	81678-16-2	Not a hazardous substance or mixture according to Regulation (EC) No. 1272/2008.
Sodium Hydroxide	1310-73-2	Corrosive to Metals (Category 1), H290 Skin corrosion (Sub-category 1A), H314 Serious eye damage (Category 1), H318
