# Supplementary figures and images for: LC–MS-Based Metabolomics Discriminates Premium from Standard Chilean cv. Cabernet Sauvignon Wines from Different Valleys
Source: Metabolites. 2021 Nov 30;11(12):829. doi: 10.3390/metabo11120829 (PMC8707972; doi:10.3390/metabo11120829)

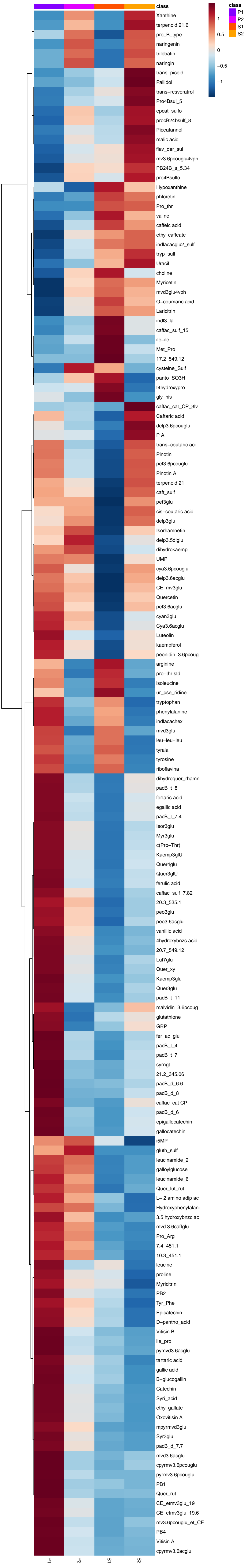

Supplement: Supplementary file 1 [file metabolites-11-00829-s001.zip › Supplementary files/Figure S4.pdf]
